# Impact of Solubilized Substances on the Techno-Functional, Pasting and Rheological Properties of Ultrasound-Modified Rice, Tef, Corn and Quinoa Flours

**DOI:** 10.3390/foods12030484

**Published:** 2023-01-20

**Authors:** Antonio J. Vela, Marina Villanueva, Grazielle Náthia-Neves, Felicidad Ronda

**Affiliations:** Department of Agriculture and Forestry Engineering, Food Technology, PROCEREALtech Research Group, University of Valladolid, 34004 Valladolid, Spain

**Keywords:** ultrasound treatment, gluten-free flours, freeze-drying and centrifugation, thermal properties, pasting properties, rheological properties

## Abstract

The modification of flours by ultrasound (US) treatments requires excess water to suspend the sample to be treated, which must be removed after treatment to recover the ultrasonicated flour. The aim of this study was to determine the influence that the water removal method has on the final characteristics of US-treated gluten-free flours (rice, brown tef, corn and quinoa). US treatment parameters were constant, and two water removal methods were studied: freeze-drying and centrifugation + drying. The elimination of water by centrifugation resulted in the loss of solubilized compounds from the treated flours, which led to important differences between the final characteristics of US-treated flours. Ultrasonication resulted in the reduction of flours’ particle size and modification of their color parameters. Techno-functional properties were modified by US treatment, where the water removal method was more influential in whole grain samples (brown tef and quinoa). Few differences were found in thermal properties among pairs of US-treated samples, indicative that the effect caused to starch was mainly attributed to ultrasonication conditions than to the drying method. The water removal method markedly influenced the pasting properties of US-treated flours, resulting in lower profiles when freeze-drying was applied and higher profiles when flours were retrieved by centrifugation. Gels made with tef, corn and quinoa presented reduced tan(δ)₁ values after sonication, while gels made with rice did not show any modification. The water removal method is a decisive step in US treatments, defining the final characteristics of the treated matter, and having a great influence in the modification attributed to ultrasonication.

## 1. Introduction

The growing gluten-free (GF) market is constantly demanding improvement in their products, by the incorporation of novel ingredients and by the application of new technologies to expand the range of applicability of the gluten-free sources. Physical modifications of starches and flours are of great potential because they involve less byproducts and no chemical residues, and for being sustainable and environmentally friendly [1]. Different physical modification methods (i.e., pulse electric field, ultrasound, microwave irradiation and high hydrostatic pressure) have been used to alter their properties and make them more suitable for different applications [2].

Ultrasound (US) treatment has shown many advantages in starch modification because of their higher selectivity and quality, reduced use of chemicals and processing time [3]. Ultrasound refers to the sound above the threshold of human hearing range (above 18 kHz) that can be classified in two categories: low-intensity, high frequency ultrasound (100 kHz–1 MHz), and high-intensity, low-frequency ultrasound (20–100 kHz), which is commonly used in the food industry [4,5]. Modifications by US need to be performed in an aqueous medium to have a homogeneous effect on the sonicated matter. The US energy is transferred to the treated matter through a process called cavitation, which is the fast generation, growth, and finally implosive collapse of bubbles in the liquid medium, generating microzones of high temperature (up to 5000 K) and pressure (up to 20 MPa) in a short time, causing changes in their structure and properties [3,6,7]. Extensive studies have been performed on starches, where the general effects of ultrasonication include changes in granule morphology, particle size, chain interactions, crystalline structure, thermal properties, swelling power, starch solubility and amylose content [2]. Research on the modification of flours by US treatments, however, is much more limited. Flours are among the main sources of carbohydrates in human consumption, commonly used in the food industry as thickener and bulking agents [8]. It is necessary to continue the research of flours as they represent more complex matrixes with a richer nutritional profile and whose industrial use is more desired.

Many factors have been suggested to influence the modification caused by US treatments on starches, such as sonication power and frequency, time and temperature of the treatment and characteristics of the treated dispersion (e.g., concentration and botanical origin of the sample) [9,10,11]. One processing factor that has not been considered when studying the effect of US treatments is the water removal method applied in the methodology. After the physical modification of starches or flours by US treatments, the liquid medium (mostly water) has to be removed in order to retrieve the modified sample. There are two water removal methods commonly reported in the literature: freeze-drying and sedimentation by centrifugation (usually followed by drying at low temperature). The different results that have been reported in the properties of starches and flours after ultrasound treatments may potentially be related to the water removal method, given that it influences the composition of the final sample (mainly due to the loss of the soluble compounds when suspensions are centrifuged). This processing step, however, has not been considered by the available literature, and the studies tend to entirely attribute the determined changes to the effects of ultrasonication.

The main objective of this research was to determine the effect that the water removal method has over the final properties of US-treated GF flours. A second objective of this study was to evaluate the influence that the botanical origin and composition of the treated flour has on the extent of modification achieved by ultrasonication. Therefore, the same US treatment was applied to four GF flours, namely rice, tef, corn and quinoa, which were subsequently dried by freeze-drying or sedimentation by centrifugation + drying at low temperature. These four GF flours were chosen given that they present different compositions and natural characteristics (i.e., cereal vs. pseudocereal, refined flour vs. whole flour) which would allow for a wider visualization of the effect of the US treatment and the drying method on different sources. Rice (*Oryza sativa* L.) is a major cereal crop, representing the staple food source for half of the world’s population. Rice flour is considered the most suitable cereal flour in GF formulations due to its soft taste, colorless, hypoallergenic properties, low levels of sodium and easy digestible carbohydrates [12,13]. Tef [*Eragostis tef* (Zucc.) Trotter] grain, originated from Ethiopia, is always consumed as a whole meal given its extremely small size, resulting in higher iron, calcium and zinc than other cereal grains, and also presents proteins with an excellent balance among the essential amino acids [14]. The modification of corn (*Zea mays* L.) starch by US treatments has been extendedly studied [2,5,7,15,16,17,18] under different treatment conditions, mainly due to its abundant availability and importance as a raw material in the food industry. Corn flour, however, has not been studied so far. Quinoa (*Chenopodium quinoa* Willd.) is a pseudocereal native from the Andes, and is considered a whole-grain GF source that is gaining increasing popularity due to its attractive nutritional quality [8,19]. Quinoa presents dietary fibers, essential amino acids, high-quality lipids, vitamins, minerals, and a large variety of bioactive compounds [8,19].

## 2. Materials and Methods

### 2.1. Materials

*Indica* rice flour was supplied by Herba Ricemills S.L. (Valencia, Spain), presenting the following composition: 13.17% moisture, 6.89% protein, 2.50% fiber, 0.69% fat and 0.52% ash. Brown tef grains were provided by CYLTEF (Villanazar, Spain), while quinoa grains were provided by Extremeña de Arroces (Caceres, Spain). Both grains were milled with a LM3100 hammer mill (Perten Instruments, Stockholm, Sweden), using a 0.5 mm opening screen size to obtain the flours. The composition of brown tef was: 8.92% moisture, 9.04% protein, 7.03% fiber, 2.61% fat and 2.75% ash. The composition of quinoa flour was: 10.47% moisture, 15.58% protein, 11.70 % fiber, 6.14% fat and 2.35% ash. Corn flour was obtained from ADAPAN Europa S.L. (Asturias, Spain) with the following composition: 12.12% moisture, 7.51% protein, 9.20% fiber, 4.42% fat and 1.14% ash.

### 2.2. Ultrasound Treatment

The ultrasound treatment was performed using a Hielscher UP400St ultrasonicator (Hielscher Ultrasonics, Teltow, Germany) with a S24d22D titanium tip. The applied frequency was 24 kHz, at 80% on-off cycle, a maximum output power of 180 W and a temperature of 20 °C, kept constant using a RA12 LAUDA water bath (Lauda-Königshofen, Germany). The applied frequency (24 kHz) lays into the high-intensity low-frequency ultrasound region commonly used for processing, preservation and extraction in the food industry due to the advantages of being clean, non-toxic and eco-friendly [4]. Flours were suspended in water at a concentration of 25% (*w*/*w*), and ultrasonication was applied for 10 min. Rice and corn samples were obtained as flours, while tef and quinoa were obtained as grains, which were milled with a LM3100 hammer mill (Perten Instruments, Stockholm, Sweden) using a 0.5 mm opening screen size to obtain the flours. Samples were agitated during treatments using a magnetic stirrer to avoid sedimentation and ensure homogeneous conditions.

After the treatments, the excess water was removed to retrieve the flours following two procedures: (a) freeze-drying, and (a) sedimentation by centrifugation followed by air drying. For the first procedure, freeze-drying was carried out using Telstar Lyoquest equipment (Terrassa, Spain), and the flours were identified with “F” (see Table 1). In the second procedure, a Beckman Coulter centrifuge model Avanti J-26 XP (Indianapolis, IN, USA) was used at 13,000 rpm for 75 min, followed by drying in a Memmert constant climate chamber model UN750 (Schwabach, Germany) at 40 °C for 24 h. These samples were identified with “C” (see Table 1). In both procedures, after removing the water, the dried flours were sieved to <250 μm. Native untreated flours were used as the control in the study and were identified with “N” (see Table 1).

### 2.3. Characterization of Soluble Compounds and Ash Content

The supernatants from centrifuged samples were placed in previously ignited, cooled and tared porcelain crucibles. The crucibles were kept at 105 °C for 24 h in an oven to determine the amount of soluble dry matter, followed by incineration in a muffle furnace at 500 °C for 24 h to determine ash content in the soluble components. Results were expressed as g soluble compounds/100 g flour, and ash content in the solubilized compounds (%). Measurements were performed in duplicate.

### 2.4. Particle Size Distribution

A Mastersizer 2000 (Malvern Instruments Ltd., Malvern, UK) equipped with a dry dispersion unit was used to determine the granulometry of the studied flours. The median diameter (D_50_) and span values [(D_90_-D_10_)/D_50_] were used as dispersion parameters according to Abebe et al. (2015) [14]. Samples were measured in triplicate.

### 2.5. Color

The color parameters of the flours were determined using a PCE-CSM 2 colorimeter (PCE Instruments, Meschede, Germany), controlled with the 3nh Color Quality Controller System (CQCS3) software Version 3.2 (Shenzhen ThreeNH Technology Co. Ltd., Shenzhen, China). Results were obtained in the CIE L* a* b* and CIE L*C* h coordinates using the D65 standard illuminant and 10° standard observer. L* indicates lightness from black (0) to white (100), a* refers to tones from green (−) to red (+), and b* from blue (−) to yellow (+). The color difference (ΔE) of the US-modified flours with respect to their control flour was calculated using the equation: ΔE = [(ΔL*)^2^ + (Δa*)^2^ + (Δb*)^2^]^1/2^. Samples were measured five times.

### 2.6. Amylose and Starch Content

Amylose and starch content were quantified using a Megazyme K-AMYL assay kit (Wicklow, Ireland), following the procedure indicated by Solaesa et al. (2020) [8]. Starch content results are referred to dry matter (dm), while amylose content results are referred to starch content. Samples were measured in duplicate.

### 2.7. Fourier Transform Infrared (FTIR) Spectroscopy

A FT-IR Nicolet iS50 spectrophotometer (Thermo Fisher Scientific, Waltham, MA, USA) coupled with a crystal diamond attenuated total reflectance (ATR) sampling accessory was used to obtain the FTIR spectra of the studied flours. Flours were equilibrated to 15% moisture content prior to measurements using a saturated humidity Memmert incubator model ICP260 (Schwabach, Germany) at 15 °C. Scanning was conducted in the range of 4000–400 cm^−1^ with a resolution of 4 cm^−1^ and the accumulation of 64 scans. Changes to the protein secondary structure were analyzed in amide I bands (1700–1600 cm^−1^) following an iterative fitting assuming Gaussian band shapes as indicated by Byler and Susi (1986) [20], using Origin2019b software (OriginLab Corporation, Northampton, MA, USA). Peaks were classified as high frequency β-sheet (1700–1690 cm^−1^), β-turns (1690–1665 cm^−1^), random structure and α-helix (1665–1640 cm^−1^) and low frequency β-sheet (1640–1615 cm^−1^) according to Fevzioglu et al. (2020) [21]. Samples were measured in triplicate.

### 2.8. Techno-Functional Properties

Water absorption capacity (WAC), water absorption index (WAI), water solubility index (WSI), swelling power (SP), foaming capacity (FC) and foam stability (FS) were determined following the methods described by Abebe et al. (2015) [14]. WAC was expressed in g H_2_O/g flour (dm), WAI in g sediment/g flour (dm), WSI in g soluble solids/100 g flour (dm), SP in g sediment/g of insoluble solids in flour, FC in mL of foam and FS in percentage of the remaining foam after 60 min (%). Emulsifying activity (EA) and emulsion stability (ES) were determined according to the method described by Kaushal et al. (2012) [13], with some modifications. The flour sample (7 g) was homogenized for 60 s in a beaker containing 100 mL of distilled water and 100 mL of Koipe Asua corn oil (Cordoba, Spain). A sample of 45 mL of the emulsion was placed into 50 mL centrifuge tubes and centrifuged at 1300× *g* for 5 min. EA was calculated by dividing the volume of the emulsified layer after the centrifugation by the volume of the emulsion before centrifugation × 100, and results were expressed as a percentage (%). The stability was determined by heating the centrifuge tubes containing the emulsions for 30 min at 80 °C, cooled to room temperature and centrifuged again at 1300× *g* for 5 min. ES was stablished as the percentage of emulsion remaining after the heating process. Results of WAC, WAI, WSI and SP were referred to dry matter (dm). Samples were measured in triplicate.

### 2.9. Thermal Properties

The thermal properties of the flours were determined using a DSC3 differential scanning calorimeter (DSC) (Mettler Toledo, Barcelona, Spain). Flour samples (~6 mg) were weighed into 40 μL aluminum pans with the corresponding amount of deionized water to reach a flour concentration of 30% (*w*/*w*). Samples were kept at room temperature for 30 min after sealing the pan to allow moisture equilibration. The scan was performed from 0 to 110 °C at a heating rate of 5 °C/min using an empty sealed pan as reference. The first scan was used to determine the gelatinization properties, while the second scan (performed after 7 days of sample storage at 4 °C) was used to evaluate retrogradation properties. The determined values were enthalpy (ΔH, J/g of starch) and the transition temperatures: onset (*T_O_*), peak (*T_P_*) and conclusion (*T_C_*), in the peaks determined in both scans. Samples were measured in duplicate.

### 2.10. Pasting Properties

The pasting properties of the studied flours were determined using a Kinexus Pro+ rheometer (Malvern Instruments Ltd., Malvern, UK) coupled with a starch pasting cell geometry set at 160 rpm, following the AACC International Method 76-21.02 Standard 2 [22]. The flour sample (3.50 g on a 14% moisture basis) was placed in the cannister with 25.0 mL of distilled water. Pasting temperature (PT) and viscosity properties [peak (PV), trough (TV), breakdown (BV), final (FV) and setback (SV) viscosities] were determined using rSpace software (Malvern Instruments Ltd., Malvern, UK). Samples were evaluated in duplicate.

### 2.11. Gels’ Rheological Properties

Dynamic oscillatory tests were performed to assess the rheological properties of gels using a Kinexus Pro+ rheometer (Malvern Instruments Ltd., Malvern, UK) with serrated parallel plate geometry (40 mm diameter) working at a gap of 1 mm and a constant temperature of 25 °C controlled by a Peltier KNX2002 C25P plate (Malvern Instruments Ltd., Malvern, UK). The gels were obtained after the measurement of the flours’ pasting properties (Section 2.10). Samples were kept in the plate for 5 min to allow relaxation before measurements. Strain sweeps were performed from 0.1 to 1000% strain at a constant frequency of 1 Hz to determine the linear viscoelastic region (LVR). Frequency sweeps were performed from 10 to 1 Hz at 1% strain, within the LVR. The obtained frequency sweep data were fitted to the potential equations indicated by Ronda et al. (2014) [12]. Samples were measured in duplicate.

### 2.12. Statistical Analysis

Analysis of variance (ANOVA) by Least Significant Difference (LSD) test at *p*-value ≤ 0.05 was performed using Statgraphics 19-X64 software (Cambridge, MN, USA). Significant differences between the native, the freeze-dried and the centrifuged samples were reported with different letters in each studied flour.

## 3. Results

### 3.1. Characterization of the Supernatant Obtained from Centrifuged Samples

**Figure 1:**
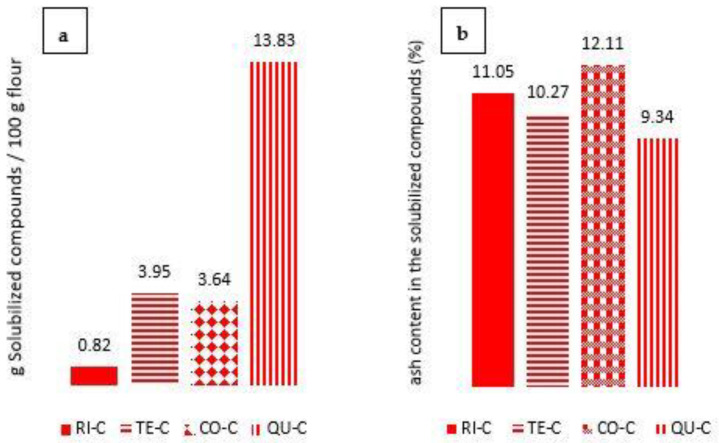
(**a**) Solubilized compounds determined in the supernatant of centrifuged samples, and (**b**) percentual ash content in said soluble fractions.

The supernatants obtained after centrifugation of the sonicated dispersions were analyzed to determine the quantity of compounds solubilized and lost after treatment, and the ash content in the extract, since these values give information about the nature of the compounds that were removed from the flours, which represent the main difference between freeze-dried and centrifuged samples. Figure 1 illustrates the differences obtained for the four studied centrifuged samples. The solubilized compounds content showed the highest values for quinoa (13.83 g/100 g flour), followed by corn and tef, and lastly rice with a much lower amount (0.82 g/100 g flour). The ash content in the native flours showed the lowest content in rice (0.52%), followed by corn (1.14%), and the highest values in the whole grain flours [quinoa (2.35%) and tef (2.75%)]. Despite these differences in the flours, the ash content determined in the solubilized components was seen to be much more similar among the four flours, indicating that most of the solubilized components (~90%) were of organic origin. These results reflect the high presence of soluble fibers and proteins in the whole grain flours (mainly quinoa), and also include the solubilized amylopectin fragments and amylose chains after partial depolymerization of starch macromolecules [23]. The absence of these solubilized compounds in centrifuged samples marks the differences that will be further analyzed in the subsequent sections.

### 3.2. Particle Size

The determined values of median particle size (D_50_) and span [(D_90_-D_10_)/D_50_] of the studied flours are presented in Table 2, and their size distribution profiles are presented in Figure S1. Results showed that ultrasonication led to a significant reduction of D_50_, and a consequent increase of the particles’ dispersion (span values) in all cases, except for CO-C and QU-C. This behavior results from particle fragmentation caused by progressive erosion due to mechanical collision and shear forces from cavitation [4,5,24,25]. Said fragmentation has been reported to generate microparticles and nanoparticles after starch chain fragmentation [18] and could lead to improved amounts of soluble compounds compared to the native flours, which are retained by freeze-drying but removed by centrifugation. This would explain the generally higher D_50_ values in centrifuged samples. In the case of the two exceptions, QU-C was not significantly different from QU-N, while CO-C was significantly higher than CO-N. The botanical origin of the sample is a factor that influences their susceptibility to fragmentation by US treatments, and by comparing the size reduction that the freeze-dried samples of these flours showed (QU-F and CO-F) with respect to their native counterparts, it can be concluded that their susceptibility was much lower than rice and tef flours. In the case of CO-C, the size increase after ultrasonication has been previously reported to happen due to agglomeration of granules in ultrasonicated starches [26,27], since the instantaneous pressure and heat generated by the rapid collapsing bubbles cause particles to aggregate [27].

### 3.3. Color

Color parameters of the studied flours are given in Table 2. Lightness (L*) was mainly increased by US treatments, which is closely related to the particle size reduction, given that smaller size particles present increased surface area that allows more light reflection [8]. Among the studied samples, tef presented the highest differences after ultrasonication, both between sonicated samples and the control (TE-F and TE-C vs. TE-N), and between sonicated samples (TE-F vs. TE-C), due to its higher pigmentation compared to the other studied flours. In rice flours, the same increase was determined regardless the drying method applied, while in corn and quinoa, CO-C and QU-F did not follow the increasing trend. CO-C presented a significantly lower L* value that could be related to lower light reflected due to particle agglomeration. The L* values of corn and quinoa flours were less susceptible to be modified by ultrasonication, in agreement with the results determined for the particle size distribution of these flours. Chromaticity was evaluated from the hue (h) and chroma (C*) obtained from a* and b* coordinates. Results showed that US treatments led to a significant reduction of C*, indicating that sonicated flours had less vivid colors in all cases. It seems like the values were lower when treated samples were dried by centrifugation, reaching values 37% (RI-C), 47% (TE-C), 18% (CO-C) and 22% (QU-C) lower than their corresponding native flour. The h coordinate increased in treated samples with the exception of CO-C and QU-C, indicating more yellowish hues than their native counterparts. This trend could be explained by the loss of soluble pigments in centrifuged samples, given that the difference between freeze-dried and centrifuged samples is particularly noticeable in tef samples, the most pigmented flour. ΔE, which combines L* a* and b*, better reflected this generalized chromaticity values reduction, clearly showing a more marked effect after centrifugation.

### 3.4. Amylose and Starch Content

The values of starch and amylose content determined for the studied samples are presented in Table 2. The starch content determined after ultrasonication differed depending on the drying method, where centrifuged samples presented higher values. These differences result from the elimination of soluble compounds by centrifugation after US treatment, resulting in flours with higher amounts of starch per gram of flour in their composition, while all of the original components are present in the freeze-dried flours. The lower protein and fiber contents in rice flour would explain the more similar values reported by RI-F and RI-C, while in the other flour with higher protein, fiber and mineral contents, the differences were more prominent.

The amylose content values were referred to the starch content in each flour. Results showed that ultrasonication generally caused a significant increase in amylose content, reflected by the results obtained in freeze-dried samples (except in rice). The increased amylose contents after ultrasonication could be explained due to molecular scission of chains by cavitation, contributing to the increase of linear fragments that are quantified as amylose [16]. The degradation of the side chains of amylopectin molecules would increase the determined amylose content, given that the rupture of the α-1,6-glucosidic bonds by ultrasound would generate some short amylose-like chains [2,7,26]. Increased amylose content has been reported after US treatments of wheat [11], maize, potato, mung bean and sago starches [16]. However, the increase is not a general effect of US, and seems to mainly depend on treatment conditions and the nature of the sample. Zhang et al. (2021) demonstrated that starches from different natures present different susceptibility to be altered by the same ultrasonication conditions, where apparent amylose content could be either increased or reduced [2]. The lower values determined in centrifuged samples could be explained as a loss of amylopectin fragments and amylose chains solubilized by ultrasonication, which were retained by freeze-drying. The ultrasonicated starches have been said to have increase solubility since low molecular weight linear fractions formed after breakage of side chains of amylopectin or cleavage of amylose chains leach easily out of the granule [10,23].

### 3.5. FTIR Spectroscopy

FTIR spectra were studied in the range of 1200–900 cm^−1^ for starch analysis, and in the range of 1700–1600 cm^−1^ for protein secondary structure analysis. The absorption bands at 1047 cm^−1^, 1022 cm^−1^ and 995 cm^−1^ have been indicated to be particularly sensitive to modifications caused by starches, and are associated with the crystalline structure, the amorphous structures and C-OH bending vibrations, respectively [3,6]. The absorbance ratio 1047/1022 is used to quantify changes in starch configuration and expresses the degree of short-range order crystallinity in the sample [9,27], while the absorbance ratio 1022/995 is assumed to represent the organization state of the double helices located inside the crystallites [3]. The values determined for the studied samples are presented in Table 3. Results showed that US treatments led to a reduction of the 1047/1022 ratio, and an increase of the 1022/995 ratio (except for tef), where the values determined for freeze-dried samples were always higher than those of the centrifuged flours. Both results suggest a higher proportion of amorphous to ordered structure zones in the sonicated flours, indicating the weakening of short-range order [1,3,25]. These results indicate the lowering of double helices in starch granules by ultrasonication, in agreement with the generally indicated statement that US treatments lead to the partial depolymerization of starch macromolecules [9,23]. Linear polymeric conformations may accumulate the applied forces of the same spatial orientation on much longer distances along the chain, being more susceptible to break [11,17].

Changes in the protein secondary structure were studied in amide I (see Figure S2), the most prominent vibrational band of the protein backbone structure [28]. The individual peaks found in amide I after iterative fitting assuming Gaussian band shapes were classified as high frequency (HF) β-sheet (1700–1690 cm^−1^), β-turns (1690–1665 cm^−1^), random structure and α-helix (1665–1640 cm^−1^), and low frequency (LF) β-sheet (1640–1615 cm^−1^) [21]; the percentual area of each component was determined (see Table 3). Results showed that ultrasonication led to greater differences in LF-β-sheet and random structure and α-helix, while fewer differences were observed in β-turns and no differences were determined in HF-β-sheet. The increase in disorder structure (random coil) and the distortion of the ordered structure (β-sheet) indicates that US treatment led to the formation of more unfolded structures and higher structural flexibility [29]. The shear forces from ultrasound cavitation disrupted the interactions between protein molecules and influenced the protein molecule internal structure [30]. The drying method did not influence the results obtained, indicative that the ultrasound treatment had a uniform effect on the soluble and insoluble protein fractions. It is worth mentioning that the applied analysis method only allows for a relative comparison between the content of the indicated components, it does not allow a protein quantification, so the fact that few differences were obtained between percentual areas in both freeze-dried and centrifuged samples does not mean that there are few differences in the amount or nature of the protein present in the flours.

### 3.6. Techno-Functional Properties

The hydration properties (WAC, WAI, WSI and SP), emulsion properties (EA and ES) and foaming properties (FC and CS) of the studied flours are presented in Table 4. WAC indicates the maximum amount of water that flours can retain when an external force is applied. Results showed that freeze-drying led to a reduction of WAC values, while centrifugation increased them (except for RI-C and CO-C). Vela, Villanueva and Ronda (2021) indicated that ultrasonication does not increase water binding sites (hydrophilic parts in carbohydrates and proteins) in rice flour, in a study where sonicated samples were dried by freeze-drying, which was similar to the trend shown by the freeze-dried samples of the present study [31]. The observed results in centrifuged samples are believed to be because the composition of the flours is affected by the drying method, presenting a higher proportion of starch in their composition (see Section 3.4). The interaction that starches have with water deeply depends on starch granule morphology and composition, and in flours, the absorption and release of water also depends on proteins, which are also modified by ultrasonication [16,23]. The starch alone in the studied flours must present more hydrophilic parts that what is presented by all of the original components combined, resulting in higher water affinity when a higher proportion of starch is present (centrifuged samples). WAI and SP values were significantly increased by ultrasonication. These properties seemed to be more influenced by the effect of US treatment than the applied drying method. It has been noted that ultrasonication is able to increase both properties in flours even at short exposure times, attributed to the physical and chemical disruption suffered by the treated samples, leading to higher water uptake and retention [9,24]. In starches, it has been generally indicated that US treatments lead to increased SP values, determined in samples from potato, millet [1], oat [10], sago [16] and sweet potato [32], in agreement with what was obtained in the studied flours. These results combined suggest a uniform effect caused by ultrasonication independently of the amount of starch in the sample’s composition (lower amounts in flours dried by freeze-drying, higher amount flours dried by centrifugation followed by air-drying and 100% in isolated starches). An increase in SP might be due to the effect of ultrasonication on the amorphous region of starch granules, allowing easier absorption of water and swelling to a greater extent than the native flours [16]. WSI values were lower in treated samples where water was removed by centrifugation, as a consequence of the fraction of soluble compounds that was eliminated along with water, resulting in US-treated flours with lower amounts of available soluble compounds at the moment of measurement. The exception observed in RI-C is believed to be because of the lower amount of soluble compounds present in the native rice flour compared to the other studied flours, being less susceptible to influence by the water removal method. CO-F and QU-F presented higher WSI values after US treatment. The structural weakening and depolymerization of starch granules after ultrasonication have been indicated as possible explanations to the increased WSI, suggesting that the molecular disruption of chains contributes to the increase in linear chains leaching outside of the swollen granules [5,23,32,33].

Proteins in flours are the main components influencing their emulsion activity and foaming capacity. Proteins, being surface active agents, can form and stabilize emulsions (EA and ES) by creating electrostatic repulsion on oil droplet surfaces [13], and can lower the surface tension at the water-air interface, thus creating foam (FC and FS) [13]. Rice flour samples did not present any EA, while the FC and FS values determined were not significantly modified by US treatment. These results are a consequence of the lowest protein content in rice flour (6.89%), compared to the other studied flours. Quinoa flour, presenting the highest protein content (15.58%), showed the highest EA and ES values, which were not affected by US treatment. Tef and quinoa flours seemed to present a higher susceptibility to be modified by ultrasonication, since their EA values showed a significant increase in sonicated and freeze-dried samples, and a significant decrease in the centrifuged samples. The increase determined in TE-F and CO-F may be related to protein solubility and conformational stability [13], which was modified by US treatments, as FTIR results indicated. FC of tef and quinoa were also improved by sonication followed by freeze-drying, while centrifugation reduced FC values in tef, corn and quinoa. The lower values reported in EA and FC of centrifuged samples are believed to be due to different protein contents in samples, depending on the applied drying method, after the loss of soluble proteins during centrifugation. Ultrasonication led to a significant increase of ES in tef flours and CO-F, while FS of all flours remained unchanged or decreased (TE-C, CO-C, QU-F) after treatment, regardless of the drying method applied.

### 3.7. Thermal Properties

The thermal properties determined for the studied flours are presented in Table 5. Thermograms showed two endothermic transitions, a first peak corresponding to starch gelatinization and a second smaller peak related to the dissociation of amylose-lipid complex and/or protein denaturation [8]. Results showed few variations in starch gelatinization enthalpy (ΔH_gel_) after US treatment, where only those determined for TE-F, TE-C and QU-C were statistically lower than their corresponding native flour. A reduction in ΔH_gel_ has been attributed to the disintegration of double helices present in the crystalline and non-crystalline regions of starch by cavitation [34,35,36], and the disruption of the amorphous regions, which facilitates the diffusion of water molecules into the starch granules and, hence, their access to the crystalline regions [25], so sonicated samples would require less energy for starch gelatinization. The fact that RI-F, RI-C, CO-F, CO-C and QU-F did not show significantly lower ΔH_gel_ values could be due to the soft treatment conditions (short time and high concentration) and lower susceptibility of those flours to be modified by ultrasonication. ΔH_gel_ results showed no significant differences between freeze-dried and centrifuged samples of the same flour (except for QU-F and QU-C), reflecting that the modification was mainly caused by US treatment and was not highly influenced by the drying method, as results are referred to the starch content in each flour. A significant reduction of ΔH_gel_ has been reported in different flours (rice [24], quinoa [19], wheat and sweet potato [4]) and isolated starches (rice [25], corn [2], potato [1], oat [10] and cassava [3]), indicative that the ultrasonication effect in the starch is rather similar despite the different chemical composition that the flours and starches may have. The onset (*T_O-gel_*) and conclusion (*T_C-gel_*) gelatinization temperatures reflect the melting temperature of the weakest and high-perfection crystallites, respectively, in the starch granules [27], while peak temperature (*T_P-gel_*) indicates the temperature when the highest energy absorption is recorded. Ultrasonication led to a reduction of all *T_O-gel_*, *T_P-gel_* and *T_C-gel_* values. The few differences observed between freeze-dried and centrifuged samples indicate that starch in the studied flours is not greatly affected by the drying method, and that the observed modified values were mainly attributed to ultrasonication. Lower gelatinization temperature values indicate that cavitation led to the structural weakening of starch configuration, allowing for greater mobility of starch polymers and promoting water accessibility to the crystalline regions [1], in agreement with the conclusion obtained from ΔH_gel_. Similar results have been reported for ultrasonicated quinoa flour [19], rice [37] and corn [7] starches in US treatments that involved freeze-drying [19] and centrifugation [37]. The enthalpy determined for the melting of the amylose-lipid complex (ΔH_am-lip_) was significantly increased by US treatments in RI-F, RI-C, TE-C and QU-C. The depolymerization of long chain amylose and amylopectin molecules caused by cavitation would generate very short amylose-like starch fragments that would increase the formation of amylose-lipid complex in US-treated samples [15,38].

A second scan was performed after 7 days of sample storage at 4 °C to evaluate the thermal transitions of retrograded starch. After ultrasonication, the enthalpy determined for the melting of recrystallized amylopectin (ΔH_ret_) was significantly increased in rice flour, reduced in quinoa flour and was unchanged in tef and corn flours. Ultrasonication led to a uniform effect on ΔH_ret_ regardless of the drying method applied (except in tef flour), varying depending on the nature of the sample. US treatment led to a significant increase of ΔH_ret_ in rice flours and a reduction in quinoa flours, while no significant modification was detected in tef and corn flours. The modified ΔH_ret_ values in rice and quinoa samples indicate that ultrasonication led to higher and lower, respectively, amylopectin retrogradation capacity. These results illustrate how influential the botanical origin of the studied sample is in determining the effect of ultrasonication over certain properties. The values determined for the dissociation of amylose-lipid complex in the second scan (ΔH_am-lip-ret_) showed no significant differences among flours, with all showing higher values than those determined in the first scan. The increased values in the second scan have been determined to be due to better conditions for complex formation after the first heating, because the leaking of amylose from starch occurs at temperatures above the starch gelatinization temperature range [39]. The transition temperatures in the second scan were not particularly affected by ultrasonication, where significant differences were only found in *T_O-ret_* of RI-F, TE-F, QU-F and QU-C, and *T_P-am-lip-ret_* of QU-F and QU-C, with respect to their corresponding native flour. 

### 3.8. Pasting Properties

**Figure 2:**
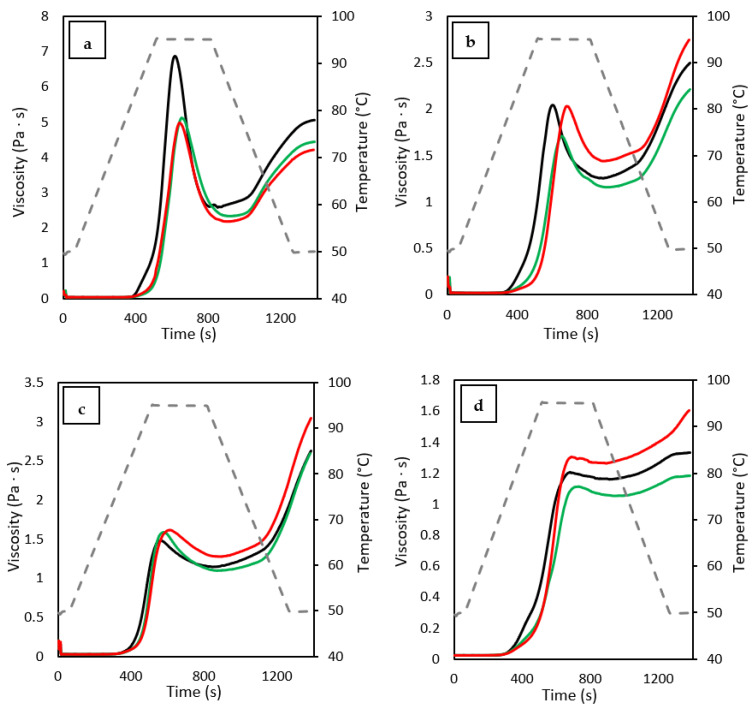
Viscometric profiles of the studied (**a**) rice, (**b**) tef, (**c**) corn and (**d**) quinoa flours. Black lines correspond to native samples, green lines to freeze-dried samples, red lines to centrifuged samples and grey discontinuous lines to the temperature of the assay.

The determined pasting properties of the studied flours are presented in Table 6, and their pasting profiles are illustrated in Figure 2. Results showed that ultrasonication led to higher pasting temperatures (PT), indicating that US treatments increased the structural resistance of starches to heat-induced swelling and rupture in water [32]. Increased PT values suggest that ultrasonication tends to increase starch crystalline perfection by strengthening intragranular bonded forces that allows starch to require more heat to achieve structural disintegration [38]. Regarding the pasting viscosity properties, there have been many contradicting reports about the modifications obtained in them after ultrasonication. While some authors indicated that US treatments increased the viscometric profiles [25,40,41,42], other authors have reported lower profiles [1,19,31,35]. The obtained results seem to point out that the lack of consensus in the available literature may be influenced by the applied water removal method, and do not entirely depend on US conditions. Pasting viscosity properties depend on different factors, such as the type of starch, the structure of amylose and amylopectin, and the presence of non-starch components due to the interaction that starch may have with them [4]. The influence of the water removal method in US treatments over the pasting properties must be directly related to the amount of soluble compounds present in the flours, given that more pronounced differences between “F” and “C” samples were observed in whole grain flours with higher protein contents [quinoa flour (15.58%) and tef flour (9.0%)]. In flours with lower protein content, softer [corn flour (7.51%)] and even no [rice flour (6.89%)] differences were observed. In quinoa and tef, the centrifuged samples showed significantly higher values of peak (PV), trough (TV), final (FV) and setback (SV) viscosities than the freeze-dried samples. In flours, viscosity development does not exclusively depend on starch; all other compounds present in the flour interact with starch and influence the pasting properties, where proteins seem to play an important role [43]. Based on the results obtained for RI-F, TE-F and QU-F, it seems like ultrasonication led to a reduction of pasting profiles, attributed to the physical damage caused to flour particles during treatment, and by changes in starch molecular structure [10,36]. The degradation of starch macromolecular chains by cavitation caused some starch molecular chains to become shorter, which would result in lower viscosity during gelatinization [7]. However, when soluble components are removed from the flour (as happens in centrifugation), the resulting flour is richer in starch, and is able to generate higher viscosity during pasting events. This effect was only not seen in RI-C, believed to be due to its lower protein, mineral and fiber contents, influencing pasting behavior to a much lower degree than the other studied flours. Breakdown viscosity (BV) provides information about the stability of the flour, indicating its ability to withstand stress and heating [31], while SV reflects its amylose retrogradation capacity [27]. Except for quinoa, the BV values determined for ultrasonicated samples where significantly reduced by US treatment, suggesting a greater resistance of starch granules to shear-thinning during cooking and strengthening stability in the hot paste [7,27,32]. The reduction of SV values determined in most of the freeze-dried samples (RI-F, TE-F and QU-F) indicates that amylose retrogradation could be depressed by US treatments, probably due to depolymerization of amylose and long-chain amylopectin [25].

### 3.9. Rheological Properties

**Figure 3:**
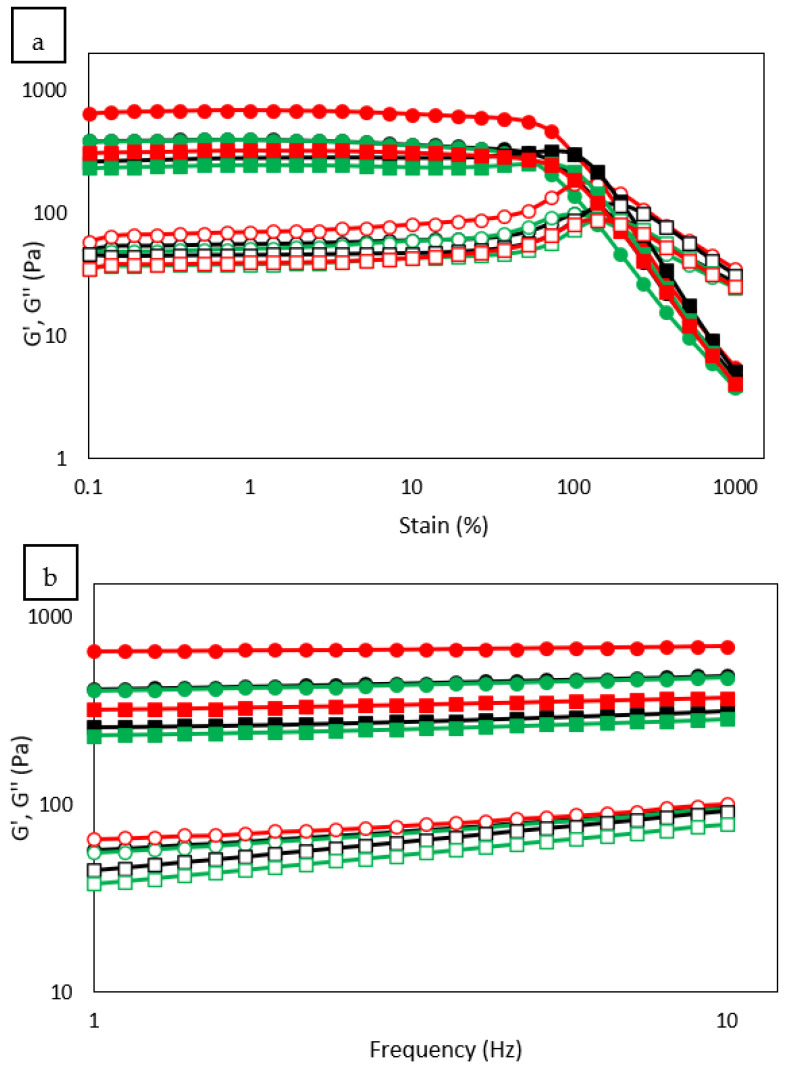
(**a**) Strain and (**b**) frequency sweeps of tef (■ □) and corn (● ○) flours. The elastic modulus (G′) is represented by solid symbols and the viscous modulus (G″) by open symbols. Black lines correspond to native samples, green lines to freeze dried samples and red lines to centrifuged samples.

The rheological properties of the gels made with the studied flours were characterized by dynamic oscillatory assays, in which strain sweeps at a constant frequency of 1 Hz determined the end of the LVR (τ_max_) and the cross over, or stress where the values of the elastic (G′) and viscous (G″) moduli are equal. Frequency sweeps were performed at a constant strain of 1%, within the LVR, and the data obtained were adjusted to potential equations. The coefficients G′_1_, G″_1_ and tan(δ)_1_ represent the elastic and viscous moduli and the loss tangent at a frequency of 1 Hz, respectively, while the exponents *a*, *b* and *c* quantify their dependence to the oscillation frequency (ω). The determined values for the strain and frequency sweeps are presented in Table 6, and the behaviors presented by tef and corn are illustrated in Figure 3. The τ_max_ and cross over values determined after US treatment showed a different behavior depending on the nature of the studied flours. In rice, no significant differences were observed, while in tef, corn and quinoa, reduced values were observed after sonication (except for CO-C), presenting lower values in centrifuged samples than in freeze-dried samples. These results reflect the fragmentation of long-chain starch molecules by ultrasound treatments, leading to the formation of weaker gels (compared to those formed by native flours) [6]. The botanical origin of the sample makes a difference in the susceptibility that they present to be altered by ultrasonication. It has been previously indicated that US treatments at different times and concentrations did not modify the cross over values of rice flour, in agreement with the results obtained for RI-F and RI-C [24]. The results of the frequency sweeps were also influenced by the nature of the flours. In rice flours, results showed that ultrasonication led to a significant reduction of G′_1_ and G″_1_, but the proportion of change of both parameters remained unchanged, thus tan(δ)_1_ was not significantly modified. In the other flours (tef, corn and quinoa), even though the values determined for G′_1_ and G″_1_ did not present a uniform trend, it could be seen that freeze-drying after ultrasonication mainly resulted in lower values, while centrifugation led to higher values. It has been indicated that the reduction of the viscoelastic moduli values by US treatments results from a combined effect of starch granule disruption and breakdown of the linear amylose molecules, since the rupture of polymeric chains leads to the straightening out of amylose molecules, and results in shorter linear amylose chains that are unable to form a consolidated viscoelastic network during gelatinization [3,9,15]. The opposite results determined in centrifuged samples are believed to be due to the higher amount of starch in their composition, with respect to their native counterpart, allowing for the formation of stronger gels which camouflage the disruptive effect of ultrasonication. The values of tan(δ)_1_ were significantly reduced after ultrasonication, except in rice flour. Lower tan(δ)_1_ values are indicative of gels with higher strength [32]. A reduction in tan(δ)_1_ values after US treatments of starches [2,9,32] and flours [24,31] has been previously reported. This reduction could be attributed to rearranged gel structures due to the straightening out of amylose and the disruption of starch granules by ultrasonication [2].

## 4. Conclusions

The natural characteristics and botanical origin of the flours influenced their susceptibility to be altered by US treatments. The water removal method greatly influenced the effect of ultrasonication on the modified flours, mainly resulting from different compositions due to the loss of soluble compounds (minerals, proteins, dietary fibers and amylopectin fragments, and amylose chains solubilized by ultrasounds) when flour samples were retrieved by centrifugation, resulting in flours with higher starch content. Ultrasonication caused a significant particle size reduction, which resulted in increased L* values, and a greater interaction with water, improving WAI and SP. Signs of partial depolymerization of starch macromolecules and a general weakening of starch structural arrangement were determined by increased values of amylose content after ultrasonication, the higher proportion of amorphous to ordered structure zones indicated by FTIR and the reduction of gelatinization transition temperatures showed by DSC. The pasting properties were markedly influenced by the water removal method, derived from the composition of the US-treated flours, where the higher starch content in centrifuged samples increased the viscometric profiles, while freeze-drying reflected the real effect of ultrasonication, mainly leading to reduced viscometric profiles. The gels’ rheological properties indicated that ultrasonication led to weaker gels that resisted lower stress before the rupture of their structure, probably related to fragmentation of starch macromolecules.

## Figures and Tables

**Table 1:** Sample identification for the studied flours.

Sample	Native	Freeze-Dried	Centrifuged
Rice	RI-N	RI-F	RI-C
Tef	TE-N	TE-F	TE-C
Corn	CO-N	CO-F	CO-C
Quinoa	QU-N	QU-F	QU-C

**Table 2:** Physical properties of native and modified rice, tef, corn and quinoa flours.

Sample	D_50_ (μm)	(D_90_-D_10_)/D_50_	L*	a*	b*	C*	h	ΔE	Amylose (%)	Starch (%)
RI-N	210 c	1.51 a	86.2 a	0.49 b	5.11 c	5.13 c	84.5 a	---	36.8 c	72.4 a
RI-F	130 a	2.40 c	88.2 b	−0.08 a	3.41 b	3.41 b	91.3 b	2.69	35.2 b	73.1 ab
RI-C	165 b	2.11 b	88.7 b	−0.06 a	3.23 a	3.23 a	91.1 b	3.18	29.5 a	74.8 b
SE	1	0.01	0.2	0.02	0.03	0.03	0.2	---	0.4	0.6
*p*-value	***	***	***	***	***	***	***	---	**	*
TE-N	121.4 c	2.44 a	59.1 a	8.1 c	11.8 c	14.3 c	55.6 a	---	39.0 b	64.8 a
TE-F	84.1 a	3.06 b	65.6 b	5.7 b	10.1 b	11.6 b	60.7 b	7.13	41.9 c	64.5 a
TE-C	95.4 b	3.16 c	72.5 c	3.8 a	6.6 a	7.6 a	60.1 b	15.00	36.8 a	70.7 b
SE	0.6	0.01	0.4	0.1	0.1	0.2	0.3	---	0.2	0.6
*p*-value	***	***	***	***	***	***	***	---	***	**
CO-N	184 b	2.09 b	77.8 b	5.82 c	31.1 c	31.8 c	79.5 b	---	37.1 a	62.5 a
CO-F	144 a	2.45 c	78.4 b	4.60 a	29.3 b	29.6 b	81.1 c	2.26	42.1 b	62.9 a
CO-C	223 c	1.80 a	75.9 a	5.07 b	25.6 a	26.1 a	78.8 a	5.87	37.8 a	70.2 b
SE	3	0.01	0.3	0.08	0.3	0.3	0.1	---	0.7	0.4
*p*-value	***	***	***	***	***	***	***	---	*	***
QU-N	161.1 b	2.23 b	75.3 a	3.57 c	13.4 c	13.82 c	75.1 b	---	22.3 b	46.2 a
QU-F	142.7 a	2.29 c	75.0 a	3.25 b	12.7 b	13.08 b	75.6 c	0.83	25.9 c	44.5 a
QU-C	161.8 b	2.06 a	76.7 b	3.05 a	10.4 a	10.81 a	73.6 a	3.35	17.2 a	54.5 b
SE	0.6	0.01	0.3	0.03	0.1	0.06	0.1	---	0.4	0.6
*p*-value	***	***	**	***	***	***	***	---	**	***

D_50_: median diameter; (D_90_-D_10_)/D_50_: size dispersion. L*, a*, b*: CIELAB color coordinates; C*: Chroma; h: hue. SE: Pooled standard error from ANOVA. Different letters in the same column of each individual sample indicate statistically significant differences between means at *p* < 0.05. Analysis of variance and significance: *** *p* < 0.001. ** *p* < 0.01. * *p* < 0.05. ns: not significant.

**Table 3:** Analysis of starch and protein secondary structure on native and treated flours using FTIR.

	Starch		Proteins Secondary Structure			
Sample	1047/1022	1022/995	LF-β-Sheet	Random Coil + α-Helix	β-Turns	HF-β-Sheet
RI-N	0.714 b	0.877 a	39.3 c	37.5 a	21.2 a	1.93 a
RI-F	0.680 a	0.894 b	31.9 a	45.0 b	21.2 a	1.91 a
RI-C	0.676 a	0.889 b	34.1 b	43.1 b	20.9 a	1.86 a
SE	0.002	0.002	0.5	0.7	0.3	0.03
*p*-value	***	***	***	***	ns	ns
TE-N	0.793 c	0.891 a	32.7 b	44.9 a	20.3 b	2.09 b
TE-F	0.699 b	0.886 a	29.0 a	46.1 b	22.3 c	2.73 c
TE-C	0.679 a	0.878 a	33.0 a	46.7 b	19.1 a	1.21 a
SE	0.001	0.005	0.5	0.4	0.1	0.08
*p*-value	***	*	**	*	***	***
CO-N	0.739 c	0.880 a	39.1 c	42.8 a	15.5 a	2.64 b
CO-F	0.729 b	0.904 b	32.2 a	48.2 b	17.3 b	2.21 ab
CO-C	0.704 a	0.882 a	34.5 b	46.8 b	16.8 b	1.90 a
SE	0.002	0.002	0.6	0.7	0.3	0.17
*p*-value	***	***	***	**	**	ns
QU-N	0.769 c	0.938 c	33.8 a	44.1 a	19.3 b	2.79 a
QU-F	0.692 b	0.919 b	32.9 a	48.9 b	16.2 a	1.97 a
QU-C	0.672 a	0.905 a	32.9 a	50.3 c	14.7 a	2.14 a
SE	0.002	0.003	0.6	0.3	0.6	0.13
*p*-value	***	***	ns	***	**	ns

LF: Low Frequency; HF: High Frequency. SE: Pooled standard error from ANOVA. Different letters in the same column of each individual sample indicate statistically significant differences between means at *p* < 0.05. Analysis of variance and significance: *** *p* < 0.001. ** *p* < 0.01. * *p* < 0.05. ns: not significant.

**Table 4:** Techno-functional properties of the studied flours.

Sample	WAC	WAI	WSI	SP	EA	ES	FC	FS
RI-N	1.31 b	7.2 a	1.6 a	7.3 a	---	---	3.0 a	31 a
RI-F	1.19 a	9.2 b	1.9 a	9.4 b	---	---	2.5 a	35 a
RI-C	1.22 a	8.8 b	2.1 a	8.9 b	---	---	3.5 a	60 a
SE	0.02	0.2	0.2	0.5	---	---	0.6	18
*p*-value	**	***	ns	***	---	---	ns	ns
TE-N	1.13 b	5.4 a	5.4 c	5.7 a	48.2 b	6 a	6.5 b	54 b
TE-F	1.01 a	7.2 b	4.5 b	7.5 b	52.3 c	29 b	7.5 b	46 b
TE-C	1.23 c	9.2 c	2.3 a	9.4 c	40.6 a	25 b	2.5 a	0 a
SE	0.01	0.1	0.3	0.2	0.7	3	0.5	3
*p*-value	***	***	***	***	***	***	*	**
CO-N	1.61 b	6.0 a	4.9 b	6.4 a	40.6 b	5.9 b	4.5 b	90 b
CO-F	1.56 a	6.8 c	6.0 c	7.3 c	45.1 c	16.5 c	4.5 b	100 b
CO-C	1.62 b	6.4 b	4.2 a	6.7 b	10.0 a	0.0 a	1.5 a	0 a
SE	0.02	0.1	0.1	0.3	0.2	0.3	0.5	6
*p*-value	ns	***	***	***	***	***	*	**
QU-N	0.92 b	8.1 a	6.2 b	8.6 a	57.7 a	55.6 a	6.0 a	83 b
QU-F	0.85 a	8.3 a	7.3 c	9.0 a	57.1 a	56.2 a	9.0 b	23 a
QU-C	1.01 c	8.2 a	5.1 a	8.6 a	57.4 a	55.4 a	4.0 a	88 b
SE	0.01	0.1	0.3	0.1	0.4	0.7	0.6	7
*p*-value	***	ns	*	ns	ns	ns	*	*

WAC = Water absorption capacity (g H_2_O/g flour dm). WAI = Water absorption index (g sediment/g flour dm). WSI = Water solubility index (g soluble solids/100 g flour dm). SP = Swelling power (g/g insoluble flour matter). EA = Emulsifying activity. ES = Emulsion stability. FC = Foaming capacity. FS = Foam stability. WAC, WAI, WSI, SP are referred to dry matter. SE: Pooled standard error from ANOVA. Different letters in the same column of each individual sample indicate statistically significant differences between means at *p* < 0.05. Analysis of variance and significance: *** *p* < 0.001. ** *p* < 0.01. * *p* < 0.05. ns: not significant.

**Table 5:** Thermal properties of the native and ultrasonicated flours.

Sample	ΔH_gel_ (J/g)	T_O-gel_ (°C)	T_P-gel_ (°C)	T_C-gel_ (°C)	ΔH_am-lip_ (J/g)	T_P-am-lip_ (°C)	ΔH_ret_ (J/g)	T_O-ret_ (°C)	T_P-ret_ (°C)	T_C-ret_ (°C)	ΔH_am-lip-ret_ (J/g)	T_P-am-lip-ret_ (°C)
RI-N	14.3 a	69.1 b	74.4 b	80.3 c	1.30 a	96.9 b	8.5 a	34.7 b	50.7 a	62.9 a	3.0 a	98.5 a
RI-F	14.2 a	68.0 a	73.2 a	78.9 b	1.58 b	96.4 ab	9.5 b	34.1 a	51.1 a	63.3 a	3.2 a	98.4 a
RI-C	14.0 a	68.1 a	73.2 a	78.5 a	1.57 b	96.1 a	9.4 b	35.0 b	50.9 a	62.7 a	2.8 a	97.9 a
SE	0.1	0.1	0.1	0.1	0.05	0.2	0.2	0.1	0.2	0.2	0.1	0.3
*p*-value	ns	**	***	***	*	ns	*	*	ns	ns	ns	ns
TE-N	13.2 b	64.8 c	71.1 c	78.8 b	0.82 a	97.1 b	6.8 ab	38.3 b	51.3 a	63.2 a	2.4 a	95.8 a
TE-F	12.3 a	64.4 b	70.7 b	77.4 a	0.95 a	95.3 a	7.4 b	35.9 a	50.6 a	63.7 a	2.2 a	96.4 a
TE-C	12.4 a	64.0 a	70.3 a	77.1 a	1.23 b	96.2 ab	5.9 a	37.7 b	50.8 a	63.5 a	1.9 a	95.9 a
SE	0.2	0.1	0.1	0.1	0.05	0.2	0.2	0.3	0.4	0.2	0.1	0.6
*p*-value	*	**	**	**	*	*	*	*	ns	ns	ns	ns
CO-N	8.8 a	62.9 c	70.7 c	77.6 b	1.2 a	96.8 a	6.3 a	38.1 a	51.2 a	62.7 a	3.5 a	89.1 a
CO-F	8.5 a	62.0 b	70.2 b	76.9 ab	1.1 a	97.7 a	6.2 a	38.8 a	50.6 a	62.5 a	3.0 a	89.5 a
CO-C	9.7 a	61.0 a	69.3 a	76.3 a	0.9 a	97.8 a	5.5 a	37.8 a	49.9 a	62.4 a	2.9 a	90.3 a
SE	0.5	0.1	0.1	0.2	0.2	0.5	0.1	0.2	0.4	0.4	0.1	0.6
*p*-value	ns	**	**	ns	ns	ns	*	ns	ns	ns	ns	ns
QU-N	16.0 b	62.3 a	70.4 b	79.6 b	1.28 a	94.6 a	1.8 b	25.2 a	46.3 a	61.0 a	3.0 a	89.5 b
QU-F	15.3 b	62.2 a	70.6 b	79.3 b	1.14 a	95.0 a	0.9 a	30.6 b	47.8 a	59.7 a	2.9 a	86.2 a
QU-C	13.0 a	61.8 a	69.5 a	78.3 a	1.46 b	94.2 a	0.9 a	31.2 b	47.6 a	60.3 a	3.4 a	86.5 a
SE	0.3	0.3	0.1	0.2	0.08	0.2	0.1	0.4	0.8	0.5	0.1	0.5
*p*-value	*	ns	*	*	*	ns	**	*	ns	ns	ns	*

ΔH_gel_ = Enthalpy of gelatinisation. T_O_-_gel_, T_P-gel_, T_C-gel_ = Onset, peak and conclusion temperatures of gelatinization. ΔH_am-lip_ = Enthalpy of the amylose-lipid dissociation. T_P-am-lip_ = Peak temperature of the amylose-lipid complex dissociation. ΔH_ret_ = Melting enthalpy of amylopectin retrograded after 7 days at 4 °C. T_O-ret_, T_P-ret_, T_C-ret_ = Onset, peak and conclusion temperatures of melting of retrograded amylopectin. ΔH_am-lip-ret_ = Enthalpy of the amylose-lipid dissociation after 7 days of sample storage at 4 °C. T_P-am-lip-ret_ = Peak temperature of the amylose-lipid complex dissociation at the second scan. All enthalpies are given in J/g starch. SE: Pooled standard error from ANOVA. Different letters in the same column of each individual sample indicate statistically significant differences between means at *p* < 0.05. Analysis of variance and significance: *** *p* < 0.001. ** *p* < 0.01. * *p* < 0.05. ns: not significant.

**Table 6:** Pasting properties of the studied flours and rheological properties of the gels made with them.

Sample	PT (°C)	PV (Pa · s)	TV (Pa · s)	BV (Pa · s)	FV (Pa · s)	SV (Pa · s)	τ_max_(Pa)	Cross over (Pa)	G₁′ (Pa)	a	G₁″ (Pa)	b	tan (δ)₁	c
RI-N	82.1 a	6.52 b	2.43 a	4.04 b	5.03 c	2.61 b	219 a	315 a	214 b	0.103 a	37 b	0.321 a	0.172 a	0.218 a
RI-F	85.0 b	5.10 a	2.25 a	2.83 a	4.48 b	2.22 a	224 a	294 a	164 a	0.107 a	28 a	0.341 b	0.171 a	0.233 b
RI-C	84.8 b	5.02 a	2.22 a	2.80 a	4.25 a	2.03 a	226 a	318 a	141 a	0.115 a	26 a	0.343 b	0.183 a	0.228 ab
SE	0.2	0.07	0.08	0.09	0.03	0.08	7	9	10	0.004	1	0.002	0.004	0.004
*p*-value	**	**	ns	**	***	*	ns	ns	*	ns	**	**	ns	ns
TE-N	78.2 a	2.04 b	1.25 b	0.78 c	2.49 b	1.24 b	231 c	322 c	254 a	0.08 a	45 b	0.314 a	0.176 b	0.23 a
TE-F	80.3 b	1.64 a	1.11 a	0.53 a	2.10 a	0.99 a	187 b	232 b	240 a	0.05 a	34 a	0.310 a	0.126 a	0.27 a
TE-C	82.1 c	2.11 b	1.43 c	0.71 b	2.75 c	1.32 b	147 a	210 a	315 b	0.07 a	39 a	0.317 a	0.122 a	0.25 a
SE	0.3	0.05	0.03	0.02	0.07	0.04	4	4	10	0.03	1	0.007	0.005	0.01
*p*-value	***	***	***	***	***	***	***	***	***	ns	*	ns	***	ns
CO-N	79.1 a	1.48 a	1.08 a	0.32 a	2.55 a	1.47 a	166 b	228 b	399 a	0.071 b	56 a	0.227 b	0.139 c	0.157 a
CO-F	80.4 b	1.56 ab	1.09 a	0.48 b	2.60 a	1.51 a	90 a	165 a	394 a	0.066 b	52 a	0.241 c	0.132 b	0.175 b
CO-C	80.9 c	1.60 b	1.29 b	0.31 a	3.06 b	1.77 b	225 c	348 c	645 b	0.030 a	65 b	0.189 a	0.101 a	0.161 a
SE	0.2	0.03	0.02	0.02	0.04	0.03	7	9	17	0.003	1	0.003	0.002	0.003
*p*-value	***	***	***	***	***	***	***	***	***	***	**	***	***	**
QU-N	75.7 a	1.21 a	1.16 b	0.04 a	1.34 b	0.176 b	24 b	53 b	276 b	0.040 a	28 b	0.261 a	0.100 c	0.22 a
QU-F	76.8 b	1.13 a	1.07 a	0.06 a	1.20 a	0.132 a	23 ab	43 a	217 a	0.040 a	17 a	0.318 b	0.079 a	0.28 b
QU-C	77.8 c	1.36 b	1.32 c	0.05 a	1.66 c	0.350 c	16 a	43 a	295 b	0.046 b	26 b	0.253 a	0.089 b	0.21 a
SE	0.2	0.03	0.03	0.01	0.03	0.004	2	3	13	0.001	1	0.009	0.002	0.01
*p*-value	***	***	***	ns	***	***	ns	**	**	**	***	**	***	**

PT = Pasting Temperature. PV = Peak Viscosity. TV = Trough Viscosity. BV = Breakdown Viscosity. FV = Final Viscosity. SV = Setback Viscosity. τmax represents the end of the LVR. G_1_′, G_1_″ and tan(δ)_1_ are the coefficients obtained from the fitting to the power law model and represent the elastic and viscous moduli and loss tangent, respectively. The a, b and c exponents quantify the dependence degree of dynamic moduli and the loss tangent with the oscillation frequency. SE: Pooled standard error from ANOVA. Different letters in the same column of each individual sample indicate statistically significant differences between means at *p* < 0.05. Analysis of variance and significance: *** *p* < 0.001. ** *p* < 0.01. * *p* < 0.05. ns: not significant.

## Data Availability

The data presented in this study are available on request from the corresponding author.

## Supplementary Materials

The following supporting information can be downloaded at: https://www.mdpi.com/article/10.3390/foods12030484/s1, Figure S1: Particle size distribution of the studied (a) rice, (b) tef, (c) corn, and (d) quinoa flours. Figure S2: Deconvolved amide I bands of the studied flours. Band assignment correspond to high frequency β-sheet (1700–1690 cm^−1^), β-turns (1690–1665 cm^−1^), random coil and α-helix (1665–1640 cm^−1^), and low frequency β-sheet (1640–1615 cm^−1^). The continuous line represents the deconvolved FTIR spectra and the discontinuous line represents the fitter curve.

## References

[1] Hu A., Li Y., Zheng J. (2019). Dual-Frequency Ultrasonic Effect on the Structure and Properties of Starch with Different Size. LWT.

[2] Zhang B., Xiao Y., Wu X., Luo F., Lin Q., Ding Y. (2021). Changes in Structural, Digestive, and Rheological Properties of Corn, Potato, and Pea Starches as Influenced by Different Ultrasonic Treatments. Int. J. Biol. Macromol..

[3] Monroy Y., Rivero S., García M.A. (2018). Microstructural and Techno-Functional Properties of Cassava Starch Modified by Ultrasound. Ultrason. Sonochem..

[4] Cui R., Zhu F. (2020). Effect of Ultrasound on Structural and Physicochemical Properties of Sweetpotato and Wheat Flours. Ultrason. Sonochem..

[5] Jambrak A.R., Herceg Z., Šubarić D., Babić J., Brnčić M., Brnčić S.R., Bosiljkov T., Čvek D., Tripalo B., Gelo J. (2010). Ultrasound Effect on Physical Properties of Corn Starch. Carbohydr. Polym..

[6] Flores-Silva P.C., Roldan-Cruz C.A., Chavez-Esquivel G., Vernon-Carter E.J., Bello-Perez L.A., Alvarez-Ramirez J. (2017). In Vitro Digestibility of Ultrasound-Treated Corn Starch. Starch Stärke.

[7] Li M., Li J., Zhu C. (2018). Effect of Ultrasound Pretreatment on Enzymolysis and Physicochemical Properties of Corn Starch. Int. J. Biol. Macromol..

[8] Solaesa Á.G., Villanueva M., Vela A.J., Ronda F. (2020). Protein and Lipid Enrichment of Quinoa (Cv.Titicaca) by Dry Fractionation. Techno-Functional, Thermal and Rheological Properties of Milling Fractions. Food Hydrocoll.

[9] Kaur H., Gill B.S. (2019). Effect of High-Intensity Ultrasound Treatment on Nutritional, Rheological and Structural Properties of Starches Obtained from Different Cereals. Int. J. Biol. Macromol..

[10] Falsafi S.R., Maghsoudlou Y., Rostamabadi H., Rostamabadi M.M., Hamedi H., Hosseini S.M.H. (2019). Preparation of Physically Modified Oat Starch with Different Sonication Treatments. Food Hydrocoll..

[11] Karwasra B.L., Kaur M., Gill B.S. (2020). Impact of Ultrasonication on Functional and Structural Properties of Indian Wheat (*Triticum Aestivum* L.) Cultivar Starches. Int. J. Biol. Macromol..

[12] Ronda F., Villanueva M., Collar C. (2014). Influence of Acidification on Dough Viscoelasticity of Gluten-Free Rice Starch-Based Dough Matrices Enriched with Exogenous Protein. LWT Food Sci. Technol..

[13] Kaushal P., Kumar V., Sharma H.K. (2012). Comparative Study of Physicochemical, Functional, Antinutritional and Pasting Properties of Taro (Colocasia Esculenta), Rice (Oryza Sativa) Flour, Pigeonpea (Cajanus Cajan) Flour and Their Blends. LWT.

[14] Abebe W., Collar C., Ronda F. (2015). Impact of Variety Type and Particle Size Distribution on Starch Enzymatic Hydrolysis and Functional Properties of Tef Flours. Carbohydr. Polym..

[15] Amini A.M., Razavi S.M.A., Mortazavi S.A. (2015). Morphological, Physicochemical, and Viscoelastic Properties of Sonicated Corn Starch. Carbohydr. Polym..

[16] Chan H.T., Bhat R., Karim A.A. (2010). Effects of Sodium Dodecyl Sulphate and Sonication Treatment on Physicochemical Properties of Starch. Food Chem..

[17] Czechowska-Biskup R., Rokita B., Lotfy S., Ulanski P., Rosiak J.M. (2005). Degradation of Chitosan and Starch by 360-KHz Ultrasound. Carbohydr. Polym..

[18] Minakawa A.F.K., Faria-Tischer P.C.S., Mali S. (2019). Simple Ultrasound Method to Obtain Starch Micro- and Nanoparticles from Cassava, Corn and Yam Starches. Food Chem..

[19] Zhu F., Li H. (2019). Modification of Quinoa Flour Functionality Using Ultrasound. Ultrason. Sonochem..

[20] Byler D.M., Susi H. (1986). Examination of the Secondary Structure of Proteins by Deconvolved FTIR Spectra. Biopolymers.

[21] Fevzioglu M., Ozturk O.K., Hamaker B.R., Campanella O.H. (2020). Quantitative Approach to Study Secondary Structure of Proteins by FT-IR Spectroscopy, Using a Model Wheat Gluten System. Int. J. Biol. Macromol..

[22] AACC (2017). Method 76-21.02. General Pasting Method for Wheat or Rye Flour of Starch Using the Rapid Visco Analyser. AACC International Approved Methods.

[23] Harasym J., Satta E., Kaim U. (2020). Ultrasound Treatment of Buckwheat Grains Impacts Important Functional Properties of Resulting Flour. Molecules.

[24] Vela A.J., Villanueva M., Solaesa Á.G., Ronda F. (2021). Impact of High-Intensity Ultrasound Waves on Structural, Functional, Thermal and Rheological Properties of Rice Flour and Its Biopolymers Structural Features. Food Hydrocoll..

[25] Yang W., Kong X., Zheng Y., Sun W., Chen S., Liu D., Zhang H., Fang H., Tian J., Ye X. (2019). Controlled Ultrasound Treatments Modify the Morphology and Physical Properties of Rice Starch Rather than the Fine Structure. Ultrason. Sonochem..

[26] Ding Y., Luo F., Lin Q. (2019). Insights into the Relations between the Molecular Structures and Digestion Properties of Retrograded Starch after Ultrasonic Treatment. Food Chem..

[27] Wang M., Wu Y., Liu Y., Ouyang J. (2020). Effect of Ultrasonic and Microwave Dual-Treatment on the Physicochemical Properties of Chestnut Starch. Polymers.

[28] Kong J., Yu S. (2007). Fourier Transform Infrared Spectroscopic Analysis of Protein Secondary Structures. Acta Biochim. Biophys. Sin..

[29] Sun X., Ohanenye I.C., Ahmed T., Udenigwe C.C. (2020). Microwave Treatment Increased Protein Digestibility of Pigeon Pea (Cajanus Cajan) Flour: Elucidation of Underlying Mechanisms. Food Chem..

[30] Yang X., Li Y., Li S., Oladejo A.O., Ruan S., Wang Y., Huang S., Ma H. (2017). Effects of Ultrasound Pretreatment with Different Frequencies and Working Modes on the Enzymolysis and the Structure Characterization of Rice Protein. Ultrason. Sonochem..

[31] Vela A.J., Villanueva M., Ronda F. (2021). Low-Frequency Ultrasonication Modulates the Impact of Annealing on Physicochemical and Functional Properties of Rice Flour. Food Hydrocoll..

[32] Wang H., Xu K., Ma Y., Liang Y., Zhang H., Chen L. (2020). Impact of Ultrasonication on the Aggregation Structure and Physicochemical Characteristics of Sweet Potato Starch. Ultrason. Sonochem..

[33] Herceg I.L., Jambrak A.R., Šubarić D., Brnčić M., Brnčić S.R., Badanjak M., Tripalo B., Ježek D., Novotni D., Herceg Z. (2010). Texture and Pasting Properties of Ultrasonically Treated Corn Starch. Czech J. Food Sci..

[34] Huang Q., Li L., Fu X. (2007). Ultrasound Effects on the Structure and Chemical Reactivity of Cornstarch Granules. Starch Stärke.

[35] Luo Z., Fu X., He X., Luo F., Gao Q., Yu S. (2008). Effect of Ultrasonic Treatment on the Physicochemical Properties of Maize Starches Differing in Amylose Content. Starch Stärke.

[36] Zuo J.Y., Knoerzer K., Mawson R., Kentish S., Ashokkumar M. (2009). The Pasting Properties of Sonicated Waxy Rice Starch Suspensions. Ultrason. Sonochem..

[37] Yu S., Zhang Y., Ge Y., Zhang Y., Sun T., Jiao Y., Zheng X.Q. (2013). Effects of Ultrasound Processing on the Thermal and Retrogradation Properties of Nonwaxy Rice Starch. J. Food Process. Eng..

[38] Babu A.S., Mohan R.J., Parimalavalli R. (2019). Effect of Single and Dual-Modifications on Stability and Structural Characteristics of Foxtail Millet Starch. Food Chem..

[39] Eliasson A.-C. (1994). Interactions between Starch and Lipids Studied by DSC. Thermochim. Acta.

[40] Cao M., Gao Q. (2020). Effect of Dual Modification with Ultrasonic and Electric Field on Potato Starch. Int. J. Biol. Macromol..

[41] Park D.J., Han J.A. (2016). Quality Controlling of Brown Rice by Ultrasound Treatment and Its Effect on Isolated Starch. Carbohydr. Polym..

[42] Pinto V.Z., Vanier N.L., Deon V.G., Moomand K., El Halal S.L.M., Zavareze E.D.R., Lim L.T., Dias A.R.G. (2015). Effects of Single and Dual Physical Modifications on Pinhão Starch. Food Chem..

[43] Meadows F. (2002). Pasting Process in Rice Flour Using Rapid Visco Analyser Curves and First Derivatives. Cereal Chem..

