# Supplementary material for: Impact of Solubilized Substances on the Techno-Functional, Pasting and Rheological Properties of Ultrasound-Modified Rice, Tef, Corn and Quinoa Flours
Source: Foods. 2023 Jan 20;12(3):484. doi: 10.3390/foods12030484 (PMC9914575; doi:10.3390/foods12030484)
Supplement: Supplementary file 1 [file foods-12-00484-s001.zip › foods-2137042-supplementary.pdf]

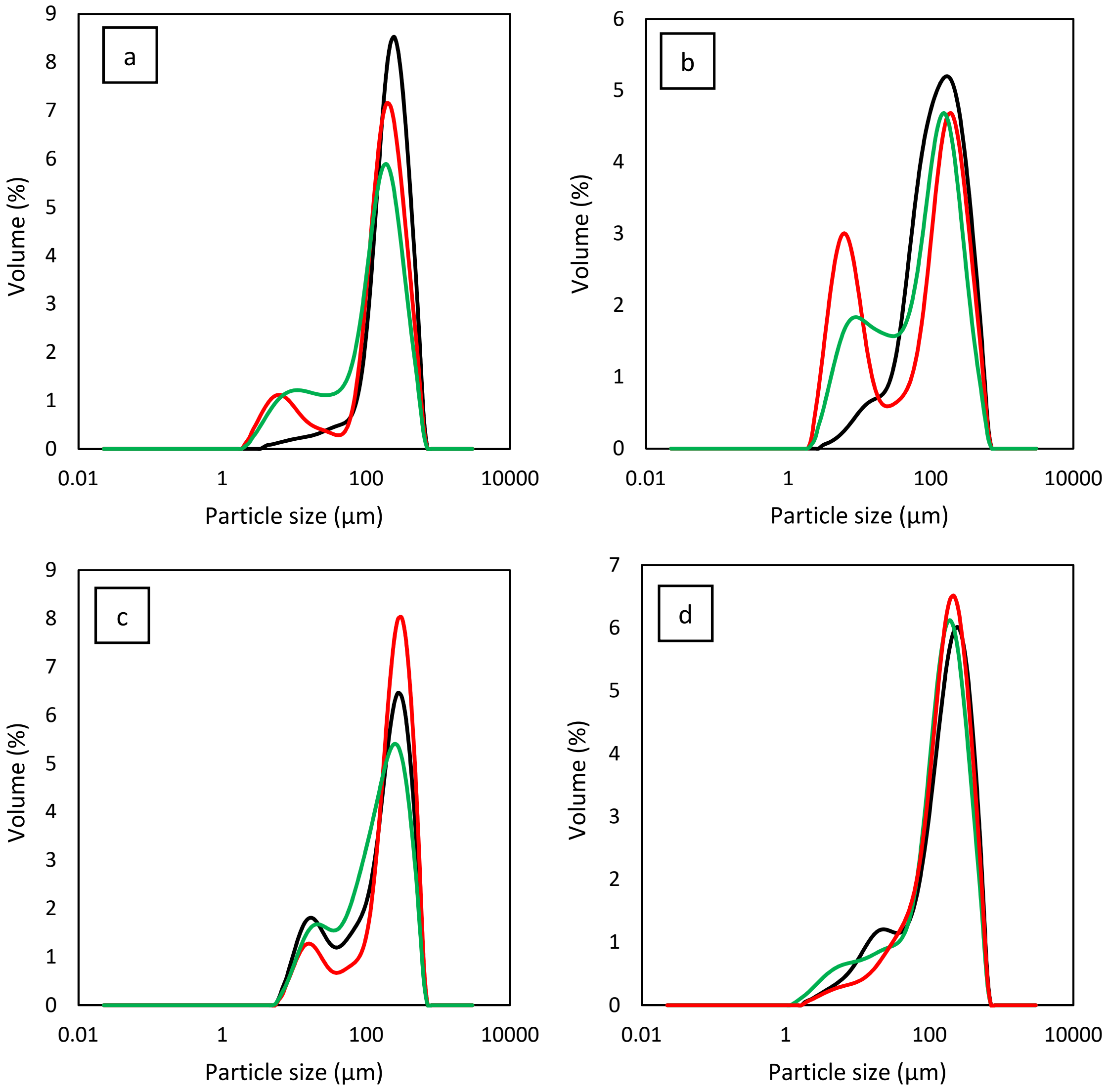

Figure S1. Particle size distribution of the studied (**a**) rice, (**b**) tef, (**c**) corn, and (**d**) quinoa flours.

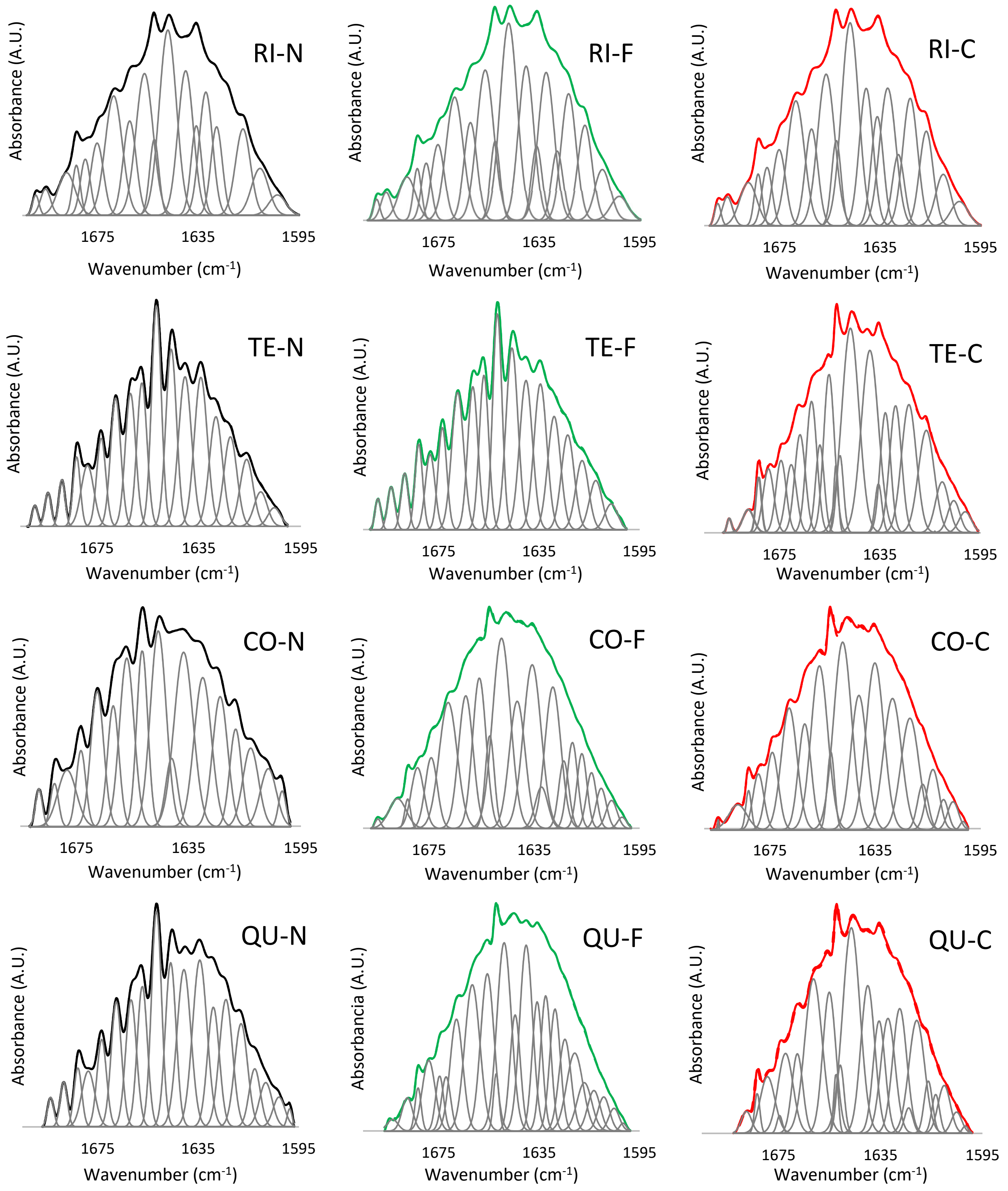

Figure S2. Deconvolved amide I bands of the studied flours. Band assignment correspond to high frequency β-sheet (1700-1690 cm^{-1}), β-turns (1690-1665 cm^{-1}), random coil & α-helix (1665-1640 cm^{-1}), and low frequency β-sheet (1640-1615 cm^{-1}). The continuous line represents the deconvolved FTIR spectra and the discontinuous line represents the fitter curve.
